# Paramedics’ experiences of barriers to, and enablers of, responding to suspected or confirmed COVID-19 cases: a qualitative study

**DOI:** 10.1186/s12913-024-11120-x

**Published:** 2024-05-29

**Authors:** Ursula Howarth, Peta-Anne Zimmerman, Thea F. van de Mortel, Nigel Barr

**Affiliations:** 1 Queensland Ambulance Service, GPO Box 1425, Brisbane, QLD 4001 Australia; 2 School of Nursing & Midwifery, Griffith University, Parklands Drive, Southport, QLD 4222 Australia; 3 Collaborative for the Advancement for Infection Prevention and Control, Gold Coast, QLD Australia; 4 Gold Coast Hospital and Health Service, Southport, QLD Australia; 5 Menzies Health Institute, Southport, QLD Australia; 6 University of Sunshine Coast School of Health, Locked Bag 4, Maroochydore, DC, QLD 4558 Australia

**Keywords:** Paramedic, Emergency medical technician, COVID-19, Pandemic, Barrier, Enabler, Ambulance service

## Abstract

**Background:**

Paramedics’ work, even pre-pandemic, can be confronting and dangerous. As pandemics add extra stressors, the study explored paramedics’ lived experience of the barriers to, and enablers of, responding to suspected or confirmed Coronavirus Disease 2019 (COVID-19) cases.

**Methods:**

This exploratory-descriptive qualitative study used semi-structured interviews to investigate Queensland metropolitan paramedics’ experiences of responding to cases during the COVID-19 pandemic. Interview transcripts were analysed using thematic analysis. Registered Paramedics were recruited by criterion sampling of staff who experienced the COVID-19 pandemic as active officers.

**Results:**

Nine registered paramedics participated. Five themes emerged: communication, fear and risk, work-related protective factors, leadership, and change. Unique barriers included impacts on effective communication due to the mobile nature of paramedicine, inconsistent policies/procedures between different healthcare facilities, dispatch of incorrect information to paramedics, assisting people to navigate the changing healthcare system, and wearing personal protective equipment in hot, humid environments. A lower perceived risk from COVID-19, and increased empathy after recovering from COVID-19 were unique enablers.

**Conclusions:**

This study uncovered barriers and enablers to attending suspected or confirmed COVID-19 cases unique to paramedicine, often stemming from the mobile nature of prehospital care, and identifies the need for further research in paramedicine post-pandemic to better understand how paramedics can be supported during public health emergencies to ensure uninterrupted ambulance service delivery.

**Supplementary Information:**

The online version contains supplementary material available at 10.1186/s12913-024-11120-x.

## Introduction

The COVID-19 pandemic disrupted healthcare globally and significantly impacted lives, including those of paramedics who perform essential frontline health care [1]. In Australia, emergency ambulance services are run/contracted by the state/territory and most qualified paramedics have a paramedicine diploma or degree and can provide advanced life support [2]. 

Prior to the COVID-19 pandemic, lessons learnt from other healthcare settings about processes of care and behaviours during disaster and emergency responses were applied to the prehospital environment [3, 4]. A recent review [5] found only nine studies that included the paramedic experience of the COVID-19 pandemic, with various foci, including leadership strategies, psychological/social wellbeing or resilience, attitudes and stressors, and knowledge and preparedness; while including two Australian studies [6, 7], none focused specifically on the experiences of paramedics in attending suspected or confirmed COVID-19 cases to examine the barriers to, and enablers of, responding to those cases. Exploring paramedics’ experience of responding under COVID-19 specific conditions may provide insights into how to increase the willingness of paramedics to respond during future public health emergencies to ensure uninterrupted ambulance service access and delivery.

This research sought to understand paramedics’ lived experience during the COVID-19 pandemic. The research question was ‘What were Queensland metropolitan paramedics’ experiences of barriers to, and enablers of, attending suspected or confirmed COVID-19 cases?’

## Methods

### Study design

An exploratory-descriptive qualitative approach [8] was applied to understand the experience of paramedics during the COVID-19 pandemic. A constructivist paradigm was chosen to explore paramedics’ experiences because it assumes there are multiple subjective realities, insider knowledge can be valuable, there is a holistic emphasis on the experience being investigated, and rich data are obtained whilst addressing context and processes [8, 9]. 

### Participant selection and setting

Registered paramedics from metropolitan south-east Queensland, Australia were invited to participate (few COVID-19 cases were occurring elsewhere at the time). Advanced Care Paramedics (ACP) and Critical Care Paramedics (CCP) in patient-facing roles with at least one year of operational experience during the COVID-19 pandemic were included. Patient Transport officers, doctors or paramedics working in supervisory roles were excluded. Criterion sampling [10] was applied to find participants with diverse education levels, age, gender and experience.

### Recruitment and data collection

The primary researcher’s management position created a potential power imbalance given the position they worked in at the time, and their previous experience in operational paramedic roles made it likely they would know participants. Consequently, they had no direct contact with participants. A research assistant (RA) was utilised to ensure participant confidentiality and to ensure they felt safe to express themselves freely. The RA had a health science doctoral qualification and invited expressions of interest via an email containing an information sheet sent by the ambulance research department. Thirty-four responses were received. After an initial screen against the inclusion criteria, the RA sent a de-identified list to the primary researcher who authorised eleven invitations to be sent out in June 2022 that maximised sample diversity. After eight interviews, no new codes were generated; one more participant was interviewed to confirm this. Four open-ended interview questions on participants’ experiences of responding to patients during the COVID-19 pandemic, and the barriers and enablers to responding to these patients were asked. The interview was piloted with a paramedic who was not part of the study; no changes to the questions were required. The RA conducted, audio-recorded and transcribed interviews (approximately 30-min in duration) in July, 2022.

### Data analysis

The research team included the primary researcher, and three doctoral qualified academics, one of whom was also a Registered Paramedic. Trustworthiness and rigour during data collection and analysis was addressed using the Lincoln-Guba framework, which underpins credibility, dependability, confirmability, and transferability [11]. During the interview and analysis phase, this included utilising a RA, member checking at the end of each interview, and researcher reflection on their own biases and preconceived thoughts after each transcript was reviewed. Researcher discussion supported rigour by identifying preconceptions the primary researcher may have that could influence data analysis [12]. Further member checking of transcripts was not deemed necessary due to the clarity of the participants’ comments.

Thematic analysis was conducted using the six-phase process outlined by Braun and Clarke [13]. The inductive method was used as the analysis was driven by the data, each participant’s language, and concepts [14], and aligns with the exploratory-descriptive qualitative approach, which focused on investigating the essence of the paramedics’ experiences during COVID-19 and remaining open to emerging themes. The transcripts were analysed by UH and all researchers discussed the coding and agreed on the themes. This discussion was informed by a range of illustrative quotes that exemplified each code.

### Ethics

Ethics approval was obtained from Royal Brisbane and Women’s Hospital Human Research Ethics Committee (Ref. no:84446) and Griffith University Human Research Ethics Committee (Ref. no:2021/819). The ambulance service approved paramedic recruitment. Participants gave informed consent.

## Results

Nine Registered Paramedics, four female and five male, aged 27–52 years (median 42; IQR = 32, 43), with 3–24 years of experience (median 8; IQR = 5, 15.5) were interviewed. Eight were ACPs, one was a CCP, all had a Bachelor of Paramedicine and two had paramedicine-related Master’s degrees. The analysis generated 26 codes and five themes: communication, fear and risk, leadership, work-related protective factors, and change.

### Communication

This theme included the codes: organisational communication, media, public health messages, and interagency communication (Table 1). Participants perceived communication - from the ambulance service, media or formal health channels – substantially impacted paramedics during the pandemic. Communication ranged from being helpful and building trust, to lacking clarity and becoming overwhelming, confusing, and frustrating.

**Table 1:** Communication

Exemplar	Codes
*“…feed information through a single point of contact, keeping everyone up to date, reduce email fatigue, and give clear directions from a clinical point of view, an operational strategic and also a* [Human Resources] *point of view to the whole state at once. Instead of it being dripped fed down from central office to regional managers down to [Station Managers]…”* (P8) *“A lot of information came through on emails, like four a day… no sort of face-to-face information sessions… some people disregarded the emails because you get… 10 emails a day and you’re like, ‘Ah, I can’t be bothered reading this’. Whereas if you had someone telling you, maybe your [Station Manager], the information would get in a bit easier.”* (P7) *“…the lack of accurate information that’s come through and I don’t know whether that patient’s not telling* [the dispatcher] *or* [the dispatcher] *not telling me …I’ve gone into houses where they haven’t told me that the whole house is infected with COVID except the patient.”* (P3) *“…there was so many protocols, and it was just forever changing from the start. It was just confusing to try and keep on top of all the new protocols.”* (P1) *“The poor man had dementia…we ended up going to [hospital] …They told us that he* [wouldn’t be admitted] *and the nursing home was just going to have to deal with* [him] *… it’s about a 45-minute drive …we ended up being with him…for three hours …he wasn’t wearing a mask. He was… getting agitated …we had to be in like close contact with him obviously to keep him calm…*[it] *was really frustrating for us because we all we could think of was* [the] *unnecessary exposure that we had to that COVID patient*.” (P1) *“…if I went to* [Hospital 1] *there was a very different set of rules than if I went to* [Hospital 2], *than if I went to a private hospital who often just rejected the patient outright …it wasn’t clear to me where the central decision-making was actually being made.”* (P2)	Organisation communication Interagency communication

### Fear and risk

The fear and risk theme included the codes: paramedic safety prioritised, physical risk to paramedic, healthcare barriers, unnecessary risk, fear of unknown, and having contracted COVID-19 (Table 2). Most indicated fear and risk influenced their personal and professional lives, with a flow on effect to patient care. Whilst mostly seen as a barrier to responding to cases, fear and risk also led to more empathetic approaches to patient care, and adherence to effective infection prevention and control practices.

**Table 2:** Fear and risk

Exemplar	Codes
*“…after I had COVID, I was feeling a lot more relaxed around COVID-positive patients.”* (P1) *“…an empathy perspective…it was easier for me to* [attend cases] *because…* [when] *I had it, I didn’t have it that bad…I was less worried if I got it again.”* (P1) *“I’ve had wild swings from initially going …I just want to get it and get it … over with …to I’m terrified of getting it …to, well we’re all gonna get it …there’s been wild swings between fear and resignation, and acceptance to back to fear again …it doesn’t just affect me, it affects people around me, and how do I keep everybody safe? And how do I keep myself safe?”* (P3) *“…having to rigorously clean the vehicle, the stretcher our equipment…”* (P6)	Contracting COVID-19 Paramedic safety prioritised Physical risk to paramedic Healthcare barriers Unnecessary risk Fear of unknown

### Leadership

The leadership theme included the codes: organisational leadership and lack of trust in organisation and government through the pandemic (Table 3). Some commented on the challenge of leadership through a pandemic, and appreciated open information-sharing, while others mistrusted decision-making and indicated the need for a consistent, visible leader.

**Table 3:** Leadership

Exemplar	Codes
*“… being able to speak to management face to face, just gives you a bit more confidence in the competence of the people … running the show.”* (P6) *“* [Organisational leadership] *was okay. It was a really, really hard job at the time …I think they did the best they could with what they had… limited information, limited resources, and directives coming from higher up that they had no control over …I think they did as best they could.”* (P7) *“I’m disappointed. …in the government for not reintroducing a mask mandate in this current wave …that’s a political decision not a health decision …that makes me sad, that they want people to be happy instead of … safe.”* (P3) *“I think the leadership was comfortable … I think we needed a face at the very beginning of this …a familiar face that led them through this challenge, just like the Chief Health Officer, that was always a single point of contact. We merged between different leaders.”* (P8)	Organisational leadership Lack of trust in organisation and government

### Work-related protective factors

Work-related protective factors covered emotional, physical, or financial support, including vaccines, leave entitlements, personal protective equipment (PPE), secure employment and comradery (Table 4). However, wearing PPE in hot, humid environments, and difficulty accessing entitlement information caused frustration and distress.

**Table 4:** Work-related protective factors

Exemplar	Codes
*“…even though it was good* [having PPE], *it was fatiguing, it was hot…it was one of those things that we knew we had to do, but we wish there was a better way.”* (P8) *“I knew they’re not going to get rid of me …a guaranteed income, which I don’t have in lots of other industries during a global pandemic …very grateful for my secure employment.”* (P3) *“… working in an environment where I’m lifting and carrying and rushing around in full PPE… I just sweat my little bum off. It’s really hard work … what we needed was breaks after PPE jobs where we could then sit down and drink water.”* (P3) *“I’ve got to go to work and do my job, loving it as much as I do*. [I] *have this feeling of responsibility and duty as it were …we obviously have a duty of care and that’s not going to change with the pandemic.”* (P6) *“Everyone was really good. I think in any station, it’s your peers that get you through. So, they were really, really good. And we would check in on each other via social media or text, and then face-to-face as well.”* (P7) *“I’ve really appreciated the information about, and support to get vaccines so I was able to get my first vaccine during work time, which I couldn’t believe …I think the* [ambulance service] *just went along, and we actually want to really support our staff to do that. So that’s been good.”* (P3)	Wearing PPE Employment Entitlements Appreciation Financial security Trust in colleagues protecting each other Access to PPE, vaccination, resources Protection with PPE

PPE – Personal protective equipment

### Change

The theme of change included the codes: adapting to their role and expectations, effect on personal life, emotional/mental health, evolution of pandemic normalised responding to cases, workload, and public reaction (Table 5). Paramedics reported issues as barriers earlier in the pandemic, but adapted as the community became highly vaccinated, their exposure to COVID-19 cases increased and it became more endemic, normalising responding to cases. Paramedics were often the first point of contact to navigate patients through the healthcare system, e.g., when patients called the ambulance service because they did not know what to do.

**Table 5:** Change

Exemplar	Code
*“…there was little dribs and drabs of information coming out daily when it first hit …we weren’t 100% sure* [of] *…the transmission rate or how quickly it appeared and how severe it could be with certain patients. And if they should go to the hospital…I guess no one really knew, so then they* [patients] *called us and …we don’t actually know either. You feel like you’re not doing* [your] *job very well.”* (P7) *“… a lot of* [General Practitioners], *as soon as it was anything which sounded like COVID, they didn’t want to know about the patient. They pushed patients away, which led to people calling the ambulance service because the ambulance service comes …always just made my mind boggle that doctors were able to choose they don’t want to see that type of patient because it’s too high risk, so it felt like we were the expendable ones.*.*”* (P9) *“ …you want to do your shopping but you couldn’t do it early in the morning. Then you had to buy toilet paper and you couldn’t because it was all gone … if you went to a COVID patient that day, you’d come home and make sure you showered and cleaned …and then the shops would be shut …your time …was a little bit more limited, because you were so focused on* [Personal Protective Equipment] *and infection control.”* (P7) *“ …we just got so blasé about it because we were going to so many of them …it was just like, yeah, righto …whatever. Instead of going one every few months we’re going to two or three a shift.”* (P4) *“…we were going to a lot of cases where the PPE was kind of unfamiliar to us and the stress levels were quite high.”* (P5) *“ …responding to patients that had a good understanding, and were being very cautious, but then their family members and friends were being belligerent …that was challenging.”* (P8)	Adapting to their role and expectations Effect on personal life Emotional/mental health Evolution of pandemic normalised responding to cases Workload Public reaction

## Discussion

Barriers to, and enablers of, Queensland metropolitan paramedics responding to suspected or confirmed COVID-19 cases were identified. Some barriers had previously been reported in studies of other healthcare workers, including communication issues, change in work practices, increased burnout, psychological distress, fear of infection to self and loved ones, lack of PPE and vaccines, and unpreparedness [15–18]. Barriers unique to the prehospital environment included ineffective communication due to the mobile nature of paramedicine, inconsistent policies/procedures between different facilities, dispatch of incorrect information, assisting people to navigate the changing healthcare system, and wearing PPE in hot, humid environments.

### Communication difficulties related to the mobile nature of paramedicine

While there can be communication issues in everyday work at the best of times, effective communication during a global infectious disease outbreak is particularly challenging due to mass media coverage, public concern, and uncertainty related to the disease [19]. Email-based communication is not always received, and communication failure can occur due to one-time message delivery, and communication fatigue [20]. In addition, media coverage, and widespread mis/disinformation created communication challenges [21]. 

Overwhelming, changing information during an outbreak is not unusual [7]. What was unique to the paramedic experience was the impact of the mobile nature of prehospital care. Attending multiple healthcare facilities per shift meant paramedics were exposed to multiple interpretations of pandemic guidance and local practices. Inconsistencies and lack of communication regarding different procedures, caused frustration, delays, and unnecessary exposure to infectious patients. This experience was confirmed in recent studies [5, 7, 22, 23]. 

One paramedic [22] attended a case where four paramedics on scene had four different oxygenation strategies, due to frequent guideline changes and the timing of accessing updates, highlighting the need for better communication strategies as an outbreak evolves.

### Increased safety risks due to receiving incorrect information from the ambulance service dispatch

Another unique communication barrier related to case dispatch. Paramedics rely on receiving correct information prior to arriving on scene to assess and mitigate risk based on what is known about the case. Miscommunication arose from the dispatcher either misunderstanding information or receiving incorrect information from the person requiring assistance, causing an increase in stress to the paramedic. Whilst case dispatch errors can occur outside of pandemic situations, the pandemic itself added an extra layer of stress in relation to paramedic safety. More stringent organisational procedures and public education are required to prevent this.

### Paramedics assisted patients to navigate the new healthcare rules

The pandemic disrupted the way healthcare was delivered and/or accessed by both health professionals and consumers [17, 24, 25]. Paramedics were affected by increased hospital waiting times, and the move to telehealth changed the types of cases they were called to [7]. Paramedics often had to navigate patients through the healthcare system to access the most appropriate help in addition to the many changes they were experiencing in their workplace and community. This indicates the need for further investigation into how paramedics can effectively assist patients when there are so many changes occurring during a pandemic, often with limited information.

### Wearing PPE in hot, humid environments, caused discomfort and fatigue

Globally, healthcare workers felt the adverse effects of wearing PPE more frequently and for longer periods [26], however, the prehospital environment created additional challenges for paramedics working in hot, humid conditions. While there is limited literature specifically on paramedics and heat-related illness when wearing PPE, during the African Ebola outbreak, the Centers for Disease Control and Prevention [27] indicated wearing PPE impairs the body’s ability to reduce body heat through sweat production, PPE holds excess heat and moisture and increases the physical effort to perform duties and the wearer can’t drink, increasing the risk of heat-related illness [27, 28]. Other common risk factors in prehospital environments include direct sun exposure, physical exertion, dehydration, and indoor heat sources at patients’ homes. Clinicians need to balance having an impermeable layer of PPE to protect against viral contamination, and the heat stress caused to the wearer [29]. While personal cooling garments are available, the effectiveness of these to decrease PPE-related heat stress has not been studied [28]. 

Healthcare workers are at increased risk of self-contamination when doffing PPE if they are experiencing PPE-related discomfort [30], have trouble completing procedures, and experience facial injuries and skin conditions, and decreased well-being and job satisfaction. These issues are particularly relevant for paramedics in hot, humid parts of Australia. Paramedic-specific research is required to better support paramedics working in these environments in full PPE.

### After contracting COVID-19, participants’ perceptions of risk reduced and empathy towards COVID-19-positive patients increased

One enabler - a decreased perception of risk and associated anxiety, and increased empathy for COVID cases after contracting COVID oneself - has not been previously reported, possibly because paramedics are used to experiencing risk in their work [31, 32]. 

This exploration of paramedics’ experiences of barriers to, and enablers of, responding to suspected or confirmed COVID-19 cases uncovered challenges unique to the prehospital field that can potentially impact service delivery. Paramedicine is often the ‘forgotten profession’ overshadowed by community and acute care, and emergency department issues [31]. While studies based on a hypothetical public health emergency and willingness to respond are helpful, there are limitations compared to exploring this phenomenon during an actual public health emergency [33]. 

### Limitations

Paramedics in non-metropolitan areas were not recruited and may have provided new insights into responding to cases in a geographically diverse state that includes logistical and resourcing challenges common in rural/remote areas. Given the specific recruitment for this study, the findings may not be transferable to other prehospital settings. Culture and personal beliefs and how these may have affected paramedics’ experience of working during a pandemic were not explored.

### Recommendations

Further research is required on methods to improve communication to paramedics, particularly cross-facility communication, and how to flag critical information changes so these changes are implemented as soon, and consistently, as possible. Strategies to mitigate the effects of PPE when worn for extended periods in hot, humid conditions should also be explored. In the meantime, supervisors should prioritise regular rehydration, breaks, and welfare checks. Research on barriers and enablers during a public health emergency from the perspective of managers, executive leadership and other ambulance service providers would provide a deeper understanding of the issues.

## Conclusion

The value of this research is that it captures Queensland metropolitan paramedics’ experience while working through the most significant public health emergency of our generation. This study uncovered barriers and enablers to responding to COVID-19 cases and thus to ambulance service delivery unique to paramedicine stemming from the mobile nature of prehospital care. It is vital that we support healthcare workers to maintain their physical and mental health, and willingly provide essential services, and that the healthcare system is ready to provide a cohesive response to public health emergencies across all sectors. This study highlights the importance of further research into paramedics in their roles.

### Electronic supplementary material

Below is the link to the electronic supplementary material.

Supplementary Material 1

## Data Availability

The datasets generated and analysed during the current study are not publicly available to protect the confidentiality of participants but are available from the corresponding author on reasonable request.

## Abbreviations

ACP: Advanced care paramedic
CCP: Critical care paramedic
COVID-19: Coronavirus disease 2019
PPE: Personal protective equipment
RA: Research assistant
