# Supplementary material for: Paramedics’ experiences of barriers to, and enablers of, responding to suspected or confirmed COVID-19 cases: a qualitative study
Source: BMC Health Serv Res. 2024 May 29;24:678. doi: 10.1186/s12913-024-11120-x (PMC11134757; doi:10.1186/s12913-024-11120-x)
Supplement: Supplementary file 1 — Supplementary Material 1 [file 12913_2024_11120_MOESM1_ESM.docx]

Interview Schedule

Research question: What are Queensland metropolitan paramedics’ experiences of barriers to, and enablers of, attending potential or known COVID-19 cases?

Demographic questions:

Qualifications

Length of time working as a paramedic

Length of time working for the QAS

Age

Gender

Interview questions:

- 1. What was your experience of responding to patients during the COVID-19 pandemic?
  2. Could you speak about your experience of barriers to responding to patients during the COVID-19 pandemic?

- 1. What would have helped you to respond to cases during the pandemic?

- 1. Could you speak about your experience of enablers of responding to patients during the COVID-19 pandemic?
